# Vitamin E as an Antioxidant in Female Reproductive Health

**DOI:** 10.3390/antiox7020022

**Published:** 2018-01-26

**Authors:** Siti Syairah Mohd Mutalip, Sharaniza Ab-Rahim, Mohd Hamim Rajikin

**Affiliations:** 1 Faculty of Pharmacy, Universiti Teknologi MARA (UiTM) Puncak Alam Campus, Selangor 42300, Malaysia; 2 Faculty of Medicine, Universiti Teknologi MARA (UiTM) Sg. Buloh Campus, Selangor 42300, Malaysia; sharaniza_abrahim@salam.uitm.edu.my (S.A.-R.); hamim400@salam.uitm.edu.my (M.H.R.)

**Keywords:** vitamin E, reproduction, antioxidant, tocopherol, tocotrienol

## Abstract

Vitamin E was first discovered in 1922 as a substance necessary for reproduction. Following this discovery, vitamin E was extensively studied, and it has become widely known as a powerful lipid-soluble antioxidant. There has been increasing interest in the role of vitamin E as an antioxidant, as it has been discovered to lower body cholesterol levels and act as an anticancer agent. Numerous studies have reported that vitamin E exhibits anti-proliferative, anti-survival, pro-apoptotic, and anti-angiogenic effects in cancer, as well as anti-inflammatory activities. There are various reports on the benefits of vitamin E on health in general. However, despite it being initially discovered as a vitamin necessary for reproduction, to date, studies relating to its effects in this area are lacking. Hence, this paper was written with the intention of providing a review of the known roles of vitamin E as an antioxidant in female reproductive health.

## 1. Vitamin E

Vitamin E was first discovered by Evans and Bishop in 1922, and it was initially denoted as an “anti-sterility factor X” that was necessary for reproduction [1]. Since then, vitamin E has been well characterized as a powerful lipid-soluble antioxidant through extensive research. The antioxidant activities of vitamin E were reported following findings on its ability to scavenge reactive oxygen species (ROS) in cellular membranes [2,3,4].

### 1.1. Sources of Vitamin E 

Vitamin E, which consists of a mixture of tocopherols (TOCs) and tocotrienols (TCTs), is available in a number of foods and plants, ranging from edible oils to nuts. Some vitamin E-containing foods include wheat, rice bran, barley, oat, coconut, palm and annatto [5,6]. Other sources include rye, amaranth, walnut, hazelnut, poppy, safflower, maize and the seeds of grape and pumpkins. Vitamin E derivatives have also been detected in human milk [7] and palm dates (*Phoenix canariensis*) [8]. Among the many sources of vitamin E, rice bran, palm oil and annatto oil have been described as the richest sources of TCTs [9]. 

### 1.2. Structure of Vitamin E 

**Figure 1:**
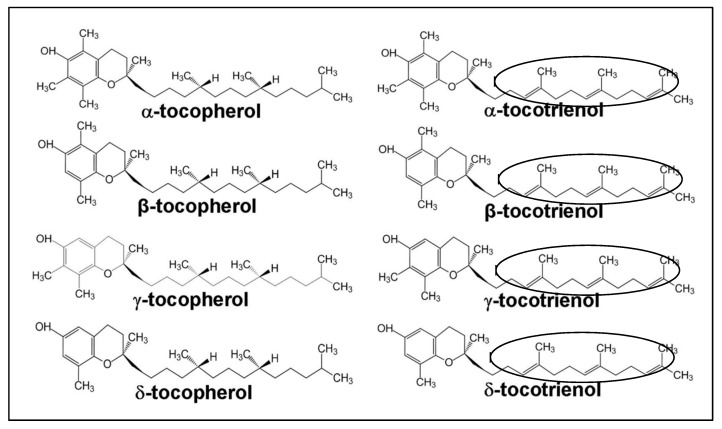
Structure differences between tocopherols (TOCs) and tocotrienols (TCTs). TOCs have saturated side chains, while TCTs have unsaturated side chains. The latter are shown by the presence of three double bonds in TCTs (circled) [14].

Vitamin E consists of a mixture of tocopherols (TOCs) and tocotrienols (TCTs) that are synthesized by plants from homogenestic acid [10]. These substances are present in eight different homologues; namely, α-tocopherol, β-tocopherol, γ-tocopherol, δ-tocopherol, α-tocotrienol, β-tocotrienol, γ-tocotrienol and δ-tocotrienol [11]. The four TOC homologues (α-, β-, γ-, δ-TOC) have a fully saturated 16-carbon isoprenoid sidechain, while TCT homologues have a similar isoprenoid chain, containing three double bonds (an unsaturated side chain). The TOC homologues are named with respect to the position and number of the methyl groups on the phenol ring. The α-, β-, γ- and δ-homologues contain three, two, two and one methyl groups, respectively (Figure 1). These structural differences and the isomerism determine the biological activity, with α-homologues being the most biologically active [12]. However, it has been reported that light, temperature, and oxygen availability could promote rancidity in vegetable oils [13]. According to a study [13], soybean oil that was stored in the dark for 56 days had increased peroxide value. In addition, its exposure to light in a 12 h light/darkness cycle over for 56 days resulted in an increase in peroxide values of around 1473%.

## 2. Reproductive Disorders: The Risk Factors

A number of risk factors contributing to reproductive- and pregnancy-related disorders have been previously reported [15,16,17]. These factors are generally categorized into two major groups: environmental and lifestyle factors. Examples of major environmental pollutants include hazardous man-made chemicals, industrial discharge, agricultural run-off, human and animal waste, municipal and domestic effluents, and spillage of vessels and oil spills [17]. Exposure to these pollutants during the time of periconceptional period (periconceptional period refers to the time of preconception, conception, implantation, placentation and embryogenesis (or organogenesis) stages of pregnancy) were reported to have adverse effects on the development of conceptus and the neonatal health [15]. These include the risks of embryonic mortality and fetal loss, intrauterine growth restriction (IUGR), birth defects, childhood diseases, premature sexual maturation and a few types of adult cancers [15]. Additionally, Rider et al. [16] also reported that exposure of conceptus to multiple environmental pollutants *in utero* during pregnancy could affect embryonic implantation and the developmental course in a cumulative dose-additive manner. 

Exposure to multicomponent mixtures of endocrine-disturbing chemicals may act as hormone mimics or antagonists, leading to the disruption of estrogen, androgen and other hormonal pathways [18]. Furthermore, exposure to multiple environmental pollutants may also result in reactive oxygen species (ROS)-induced oxidative stress (OS) [19,20,21]. The presence of high levels of OS may be a risk factor for a number of pregnancy-related disorders, such as embryonic mortality, early spontaneous abortion, IUGR, fetal death, premature delivery and low birth weight [22,23]. 

Lifestyle factors represent another category of major risk factors for reproductive and pregnancy-related disorders. Unhealthy lifestyle behaviors, including cigarette smoking, alcohol consumption, and/or drug abuse, have negative impacts, particularly on female fertility [24,25]. The underlying mechanism of the developmental defects following these unhealthy lifestyle behaviors is mainly a result of an increase in ROS production and associated OS-induced cellular damage [26]. There are also extensive epidemiological studies which have reported on a number of factors such as exposure to tobacco and alcohol, diet, stress, and gestational diabetes as the factors influencing fetal development including miscarriages [27,28,29]. 

Much evidence-based epidemiological, clinical, and experimental data on the adverse effects of cigarette smoking on female reproductive health has been reported [30]. The effects of smoking on steroidogenesis, folliculogenesis embryo transport, endometrial receptivity, endometrial angiogenesis, uterine blood flow, and uterine myometrium, all of which are related to delayed or failed implantation and pregnancy loss, have been reported. This is in line with an animal study on the effects of alcohol on reproductive health and pregnancy that indicated that prenatal exposure to ethanol in rats induced hypothalamic OS and neuroendocrine alterations in offspring [31]. Furthermore, excess ethanol administration to pregnant mice [32] and rats [33] caused disturbances in embryogenesis and increased the rate of malformations and fetal death by inducing high levels of OS. Medication use or drug abuse during pregnancy has also been associated with OS [34,35]. Phenytoin [36], thalidomide [37], valproic acid [38], almokalant, dofetilide, cisapride and astemizole [39] are the examples of identified medical drugs known to induce OS and affect the embryonic development leading to birth defects. 

To explain further, maternal smoking during pregnancy has been widely recognized as one of the most common factors of reproductive- and pregnancy-related disorders. Cigarette smoke contains a complex mixture of numerous toxic constituents including nicotine, polycyclic aromatic hydrocarbons, and cadmium [30,40]. The different constituents of the mixture cause an increased level of OS and adversely affect the cell proliferation and differentiation during embryonic development in pregnant female smokers [41]. This is supported by studies on the effects of maternal smoking during pregnancy, showing that cigarette smoking is associated with spontaneous abortion [42], placenta previa and placental abruption [43,44,45], low birth weight and preterm birth [46,47,48], stillbirth [49,50] and sudden infant death syndrome (SIDS) [51]. 

One of the most important cigarette smoke constituents, nicotine, has been reported to reduce fertility during adulthood in women [52]. In addition, cotinine (a metabolite of nicotine), cadmium, and benzo[a]pyrene have also been detected in the follicular fluid of smoking women [53,54,55], suggesting that the chemicals present in cigarette smoke can accumulate in the ovary. The results of these studies suggested that smoking women might develop impaired fertility, resulting from the combination of deteriorated oocyte function and viability [53,56,57]. 

In addition, laboratories studies have indicated that maternal exposure to cigarette smoke or cigarette smoke condensate (CSC) for 4 weeks results in the increased oocyte fragmentation or delayed fertilization, thus reducing the embryonic development to blastocysts in vitro [58]. Additionally, fragmented oocytes also showed increased production of ROS. Another study on the effects of nicotine on early embryogenesis in murine embryos reported that embryos treated with 3–6 µM of nicotine were smaller than control embryos [59]. Meanwhile, embryos treated with 6 µM of nicotine showed severe defects in the posterior trunk, resembling caudal dysplasia [59]. In addition, excessive apoptosis was also observed in the deformed structures and this was associated with the increased levels of ROS [59]. Nicotine exposure during fetal and neonatal development was also reported to cause reduction in fertility, dysregulation in ovarian steroidogenesis, and alterations in follicle dynamics in female offspring [60]. This has been further supported by another study which reported that treatment with 5 mg/mL nicotine beginning from day 1 of pregnancy throughout gestation decreased the pregnancy rates by 33.3% in Sprague-Dawley rats [61]. Another study by Rajikin et al. [62] reported that the ultrastructure of oocytes from nicotine-exposed mice showed a non-spherical shape with rough surface and torn *zona pellucida*. In addition, treatment with 5 mg/kg nicotine for 30 days increased the apoptosis rate in oocytes [63]. Meanwhile, productions of hatched blastocysts were decreased following injection with 1 mg/kg and 3 mg/kg of nicotine, and embryonic development ceased at the morula stage following exposure to 5 mg/kg of nicotine [64]. This was in line with the work of Phoebe et al. [65], which showed that after 12 weeks of cigarette smoking (directly to the lungs) in mice, the retrieved oocytes had a significantly thicker *zona pellucida*, and also shorter and wider meiotic spindles. 

### Oxidative Stress (OS) as One of the Risk Factors in Reproductive Disorders 

Oxidative stress (OS) is widely recognized as the key element in the pathogenesis of most of the diseases [66], and occurs when there is an imbalance in the presence of antioxidants and pro-oxidants [20,22,67]. Excess pro-oxidants induce OS by either generating reactive oxygen species (ROS) or by inhibiting antioxidant systems [68]. ROS are highly reactive and unstable. They acquire electrons from nucleic acids, lipids, proteins, carbohydrates, or any other nearby molecule causing a string of chain reactions to become stable. These chain reactions result in cellular damage and diseases [69]. 

In the female reproductive system, ROS can impair cellular functions and subsequently interrupt intracellular homeostasis and furthermore lead to cell damages. The presence of excess ROS can influence early embryonic development through modification of the key transcription factors that modify gene expressions [70]. High concentrations of ROS in the female reproductive tract could also negatively affect the fertilization of oocytes and cause inhibition of embryonic implantation [71,72]. Additionally, earlier studies reported that OS is involved in defective and retarded embryonic development due to OS-induced cell-membrane damage, DNA damage, and apoptosis [73,74]. Apoptosis results in the formation of fragmented embryos which have limited chances of implantation and growth [75]. 

Previous studies on the effect of OS during the periconceptional period have shown that the placenta could be the key source of OS because of the high metabolic rate and increase in the mitochondrial activities [76,77]. During the first trimester, placental tissues contain low concentrations and activities of principle antioxidant enzymes including catalase, glutathione peroxidase, and superoxide dismutase. This condition may expose the embryonic trophoblast cells to oxygen-mediated damage [78]. An earlier study reported that due to the increase in the oxygen tension during the onset of maternal arterial flow during the beginning of second trimester, a burst of OS was observed in the placenta [79]. The study suggested that this oxidative injury could adversely impair placental remodeling and functions that would subsequently affect the course of gestation [79]. This was further supported by Jauniaux et al. [80], who found that high production of ROS and reduced antioxidant defense capability might cause the developing fetus to be exposed to increased OS. 

According to other reports, macromolecule damage mediated by OS has been suggested as a mechanism of thalidomide-induced embryopathy and other embryopathies [81,82]. This suggestion was supported by an experimental finding on untreated pregnant mutant mice with a hereditary glucose-6-phosphate dehydrogenase (G6PD) deficiency that resulted in decreased litter size at birth and increased pre- and post-natal (pre-weaning) death. G6PD is a cytoprotective enzyme for OS. This result indicated that a physiological level of endogenous OS due to a dysfunctional G6PD enzyme during development can cause embryopathy that might lead to both infertility and death [83]. 

Oxidative stress in the female reproductive system is generally reported in most reproductive- and pregnancy-related disorders. For instance, OS has been associated with endometriosis. Although there is no established information on the involvement of OS in endometriosis, a number of studies have reported on the increased level of OS markers in patients with endometriosis [84,85,86,87,88,89,90]. Additionally, OS also has been reported to be involved in cases of spontaneous abortion and idiopathic recurrent pregnancy loss [66,78,80,91], unexplained infertility [92,93,94], preeclampsia [95,96,97], intrauterine growth restriction (IUGR) [98,99] and preterm labor [100,101,102,103]. 

## 3. Antioxidants and Their Roles in Reproductive Disorders 

Antioxidants regulate the overproduction of ROS. They are present in two types, enzymatic and non-enzymatic forms. Enzymatic antioxidants, including superoxide dismutase (SOD), catalase, glutathione (GSH) peroxidase and glutathione (GSH) reductase are also known as natural antioxidants or endogenous antioxidants [66,104]. The non-enzymatic antioxidants, also known as exogenous antioxidants, are obtained from dietary fruits and vegetables. These include taurine, hypotaurine, β-carotene, selenium, zinc, vitamin C and vitamin E [66]. 

The roles of antioxidants during the periconceptional period have been previously reported [105,106]. Endogenous antioxidants play important roles within the placenta as well as in the protection of trophoblast cells from OS [106]. It has been reported that SOD has a primary role in cellular protection, metabolizing two molecules of superoxide (O_2_^−^) to produce hydrogen peroxide (H_2_O_2_) and molecular oxygen (O_2_). Meanwhile, catalase (predominantly located in the peroxisomes) catalyzes the conversion of H_2_O_2_ to O_2_ and water (H_2_O). GSH peroxidase and GSH reductase are involved in oxidizing glutathione peroxides by removing H_2_O_2_ and lipid hydroperoxides [106]. 

Another antioxidant system that is highly available in the placental cells is the thioredoxin system [105]. This system consists of three antioxidant enzymes; namely, thioredoxin peroxidase, thioredoxin, and thioredoxin reductase. Thioredoxin peroxidase catalyzes the conversion of H_2_O_2_ and alkyl hydroperoxides to H_2_O and corresponding alcohols. This reaction results in the oxidation of thioredoxin peroxidases to an inactive state requiring reduction by thioredoxin [105]. Thioredoxins have been reported to be involved in a number of cellular functions, including cell growth [107], reduction of thioredoxin peroxidase [105], inhibition of apoptosis through the binding of apoptosis signal-regulating kinase-1 (ASK-1) [108], and the supply of electrons for the synthesis of deoxyribonucleotides by ribonucleotide reductase [109]. 

Exogenous antioxidants, in line with their endogenous counterparts, also play a prime role in cellular defense against OS. The effects of maternal taurine deficiency, including growth retardation of the offspring, impaired perinatal development of the central nervous and pancreatic endocrine systems, impaired glucose tolerance, and vascular dysfunction, were reported by Aerts and Van [110]. Another exogenous antioxidant, zinc (Zn) is used in assisting the fetal brain development and also as an aid to the mothers in labor [111]. According to one study, an early and progressive decline in serum Zn occurs during pregnancy, and therefore the capacity for metabolic adaptation of pregnant mothers may be limited if the maternal Zn status is poor [112]. This is supported by a meta-analysis study on zinc-supplementation in women, which resulted in 14% of reduction in premature delivery [113]. 

Vitamin C acts as a reducing agent to protect cells against the adverse effects of OS [114]. Zhang et al. [115] reported that pregnant women who consumed vitamin C at levels lower than the recommended daily allowance (85 mg) had a 2-fold higher risk of developing preeclampsia, suggesting the importance of vitamin C supplementation in pregnant women. One randomized controlled clinical trial on patients with luteal phase defects reported that pregnancy rates were higher in the group supplemented with vitamin C (750 mg/day) than in the control group (no treatment) [116]. Another double-blinded, placebo-controlled pilot study on the effect of supplementation containing vitamin E, iron, zinc, selenium and L-arginine resulted in an increase in ovulation and pregnancy rates [117]. 

Maternal (preeclampsia, abortion, and hypertension) and neonatal outcomes following antioxidant supplementation for 8 to 12 weeks in pregnancy for women with low antioxidant status were reported by Rumiris et al. [118]. This study was a randomized, double-blind, placebo-controlled trial of daily antioxidant supplementation. The supplementation included vitamins A (1000 international unit (IU)), B6 (2.2 mg), B12 (2.2 µg), C (200 mg), and E (400 IU), folic acid (400 µg), N-acetylcysteine (200 mg), Cu (2 mg), Zn (15 mg), Mn (0.5 mg), Fe (30 mg), Ca (800 mg), and selenium (100 µg). Meanwhile, the control subjects were given ferum (30 mg) and folic acid (400 µg). Results from this study indicated that antioxidant supplementation was associated with better maternal and perinatal outcomes in pregnant women with low antioxidant status as compared to control supplementation with iron and folate alone [118]. 

In addition, vitamin E functioning as a chain-breaking antioxidant was reported to protect cellular membranes against ROS, for example through defending polyunsaturated fatty acids (PUFAs) from auto-oxidation [119]. Antioxidants such as vitamin C and vitamin E have been reported to be efficient, and their uses in reproductive- and pregnancy-related disorders have been the subject of significant clinical trials [120]. For instance, a randomized clinical trial was conducted from January 2007 to February 2008 at the Women’s Hospital of Tabriz University of Medical Sciences, Iran. This study was conducted in response to the inadequate available evidence about the role of supplementary vitamin E in normal pregnancy, and assessed the potential benefit of vitamin E supplementation on health in pregnancy [121]. This trial involved 104 pregnant women who were treated with vitamin E supplementation, and 168 women (control) who were not treated with the supplementation. Treated women were administered 400 IU vitamin E from week 14 to the end of the pregnancy. The study result indicated a non-significant relationship between supplementation and maternal and perinatal outcomes and birth weight, in which preeclampsia was reported to occur in 1% of treated women as compared to 1.78% of women in the control group. From these results, the authors concluded that the administration of supplementary vitamin E starting from the second trimester of pregnancy did not show any risks with respect to pregnancy outcomes and the occurrence of preeclampsia [121]. 

This is also supported by earlier studies on the possible beneficial effects of supplementary vitamin E during pregnancy, which investigated the changes in vitamin E levels in normal versus problematic pregnancies. Oxidative stability of vitamin E levels was shown to increase in maternal blood during normal pregnancies [122]. Moreover, it has also been shown that vitamin E requirements may increase in some circumstances, such as in smoking during pregnancy [123]. In a comparative study between abnormal and normal pregnancies, the mean levels of vitamin E were reported to increase from 12.9 μg/mL in early pregnancy to 22.5 μg/mL at term in normal pregnancies. However, vitamin E levels were lower than in normal pregnancies at the corresponding gestational age in abnormal pregnancies [124]. Another study by Tamura et al. [125] on 289 pregnant women in Birmingham, United Kingdom reported that there were no significant associations between vitamin E serum concentrations and pregnancy outcomes. All of these reports suggest that vitamin E is essential for normal and healthy pregnancy, and supplementation of vitamin E does not cause any detrimental effects on pregnancy outcomes. 

Another recent study was conducted on the effects of vitamin E on the treatment outcomes of women with unexplained infertility who were undergoing controlled ovarian stimulation and intrauterine insemination (IUI) [126]. The study was conducted between June 2011 and December 2011 in Zekai Tahir Burak Women’s Training and Research Hospital, Reproductive Endocrinology and Infertility Department, Ankara, Turkey. The study groups were divided into Group A (*n* = 53) and Group B (*n* = 50). Group A underwent controlled ovarian stimulation with clomiphene citrate with vitamin E administration at 400 IU/day, while Group B (control) underwent ovulation induction without the vitamin E administration. The results of the study showed that the difference in the endometrial thickness on the day of human chorionic gonadotropin (hCG) administration was significant between the two groups; however, there was no significant association observed between vitamin E administration and implantation and pregnancy rates. Based on these results, it was concluded that vitamin E administration could improve endometrial response in women with unexplained infertility through the antioxidant and anticoagulant effects. Vitamin E may also modulate the anti-estrogenic effect of clomiphene citrate. Moreover, the issue of thin endometrium in patients may also be improved by vitamin E [126]. 

As discussed above, vitamin E has been proven to be beneficial in pregnancy and neonatal health. This is in line with previous studies that reported, for instance, plasma α-tocopherol concentrations that is below than 12 mmol/L are associated with increased infection, anemia, growth retardation and poor pregnancy outcomes in both mothers and infants (reviewed in [127]). These problems occur mainly because when low dietary amounts of α-tocopherol are consumed, the requirements for tissue α-tocopherol will exceed the available amounts, resulting in increased damages of the tissues [127]. 

### Vitamin E as an Antioxidant in Female Reproduction: The Reported Studies 

Following the first publication by Evans and Bishop [1], a later report discussed the role of vitamin E in reproduction after observations in which a vitamin-E deficient diet resulted in uterine discolorations in rats [128]. Decades later, research on the role of vitamin E in reproductive physiology was re-initiated, and it was reported to have beneficial effects against stress-induced oxidative stress (OS) [129,130,131,132,133]. 

In another study, a population of women suffering habitual abortion was observed to have high levels of lipid peroxidation and decreased levels of plasma vitamin E [129]. Another study conducted in Egypt also reported that vitamin E to be a key missing micronutrient in children with stunted growth [130]. The study showed that 78.2% of children with stunted growth had vitamin E deficiency, where plasma α-TOC concentrations were recorded at 7.7 μmol/L as compared to 14.1 μmol/L in control (normal) children. In addition, a recent report by [131] also indicated that vitamin A, E, and D deficiencies were very common in very-low-birthweight Tunisian neonates and were associated with preeclampsia. 

In more detailed experiments using in vivo laboratory animal models supplemented with palm-tocotrienol rich fractions (TRF), Mokhtar et al. [61] reported that co-administration with 5 mg/kg body weight (bw) of nicotine and 60 mg/kg of tocotrienol-rich fraction (TRF) increased the rates of pregnancy to 83.3% in rats, compared to those treated with nicotine alone, who had pregnancy rates of 33.3%. About 25.7% of the embryos developed into 2- and 4-cell stage in rats treated with both nicotine and TRF [61]. In addition, there was also a report stating that supplementation with γ-TCT in nicotine-induced mice reduced the detrimental effects of nicotine on the ultrastructure of the oocytes [62]. Another study conducted using concurrent treatment with corticosterone (CORT) and TCT reported that the numbers of abnormal embryos were reduced following supplementation with 90 mg/kg and 120 mg/kg of TCT [134]. Meanwhile, co-administration with γ-TCT improved the embryonic development in nicotine-induced mice [64]. Moreover, as reported in more recent findings, using co-incubation in media supplemented with γ-TCT and hydrogen peroxide (H_2_O_2_), γ-TCT improved the development of porcine embryos through modulation of the apoptotic BCL-XL and BAX genes [135]. The beneficial effects of TCTs were also supported by the reports on the concomitant supplementation of TRF with the anti-cancer prodrug, cyclophosphamide (CPA) on ovarian cells, which was reported to provide protection against OS-induced apoptosis in the ovaries [132,133]. 

An earlier study using supplementation with annatto-TCTs in pregnant Wistar rats reported that no adverse effects, no increase in embryo lethality and no reduction in fetal body weight were observed [136]. These findings were in line with our recent findings, together with our observations on the anti-survival effects of annatto-delta tocotrienol and soy alpha-tocopherol on the preimplantation embryos of nicotine-treated female mice [137,138,139,140]. Furthermore, a recent study also reported that annatto-TCTs suppressed cell growth in human prostate cancer cells through inhibition of the *Src* and *Stat3* genes [141]. 

In addition to the studies in human and laboratory animals, the benefits of vitamin E have also been studied in domestic animals. An earlier study using the culture of bovine embryos (embryos were derived from the in vitro matured-and-fertilized oocytes) with vitamin E, vitamin C, and ethylenediaminetetraacetic acid (EDTA) showed that more zygotes were developed to the expanded blastocyst stage in culture medium containing 100 µM of vitamin E compared to the control medium. The development to the early, expanded, and hatched blastocyst stages were also lower in the culture medium supplemented with both vitamin E and C, compared to the medium supplemented with vitamin E alone [142]. Moreover, the in vitro-produced embryos were cultured for 5.5 days in medium with or without 100 µM of vitamin E and were non-surgically transferred to recipient cows. After 7 days of transfer, the embryos were non-surgically collected, and the results indicated that embryos cultured with vitamin E were approximately 63% larger in surface area than in the control embryos [142]. Another study by [143] also reported on the effects of antioxidants such as beta-mercaptoethanol (beta-ME) and vitamin E where both suppressed oxidative damage and improved the developmental ability in the porcine embryos.

The beneficial effects of vitamin E were also studied in buffalos. A study was conducted to find whether the supplementation of vitamin E in the culture medium could ameliorate the developmental competence of preimplantation buffalo embryos. The study results indicated that under the culture condition of 20% of O_2_ level, the frequency of blastocyst formation and the total cell count were enhanced, and the formation of comet tail (DNA fragmentation) was significantly reduced following supplementation with 100 μM of vitamin E [144]. Another similar study was also conducted in sheep with the aim of determining the effects of α-TOC supplementation of the oocyte maturation media and embryo culture media on the yield of the embryos. Findings from the study showed that supplementation with 200 µM of α-TOC in the embryo culture medium at 20% of O_2_ level significantly increased the rates of cleavage, formation of morula and blastocysts, and the total cell number of blastocysts, as compared to the control groups [145].

## 4. Conclusions

Vitamin E has received much attention in recent years due to its ability to improve reproductive health. As discussed in the present paper, vitamin E has been reported to exert beneficial effects as an antioxidant against the reproductive disorders. Hence, it is highly recommended for women to consume vitamin E regularly, especially those who are in their reproductive age. However, available study reports on the effects of vitamin E on reproduction, pregnancy, and preimplantation embryonic development are still lacking. Many future studies are necessary in order to gain a greater understanding of the antioxidative role of vitamin E, especially with respect to female reproductive health.
